# Characteristics of asymptomatic COVID-19 infection and progression: A multicenter, retrospective study

**DOI:** 10.1080/21505594.2020.1802194

**Published:** 2020-08-12

**Authors:** Chao Yu, Miao Zhou, Yang Liu, Tinglin Guo, Chongyang Ou, Liye Yang, Yan Li, Dongliang Li, Xinyu Hu, Li Shuai, Bin Wang, Zui Zou

**Affiliations:** a Department of Respiratory and Critical Care Medicine, Naval Hospital of Eastern Theater of PLA,Zhoushan, Zhejiang, P. R. China; b No.5 Department of Infectious Diseases, Guanggu District, The Maternal and Child Health Hospital of Hubei Province, Wuhan, Hubei, P. R. China; c Department of Anesthesiology and SICU, Xinhua Hospital, School of Medicine, Shanghai Jiaotong University, Shanghai, P. R. China; d Department of Anesthesiology, Changzheng Hospital, Second Military Medical University, Shanghai, P. R. China; e No.2 Department of Infectious Diseases, Wuhan Huoshenshan Hospital, Wuhan, Hubei, P. R. China; f Department of Orthopedics, Ningxiang People’s Hospital, Changsha City, Hunan, P. R. China; g Department of Tuberculosis, North Ward 5, Wuhan Jinyintan Hospital, Wuhan, Hubei, P. R. China; h Department of Hepatobiliary Medicine, The 900th Hospital of the People’s Liberation Army Joint Service Force, Fuzhou, Fujian, P. R. China; i No.8 Department of Infectious Diseases, Taikang Tongji (Wuhan) Hospital of Hubei Province, Wuhan, Hubei, P. R. China; j Graduate School, Hebei North University, Zhangjiakou, Hebei, P. R. China; k Department of Oncology, Changhai Hospital, Second Military Medical University, Shanghai, P. R. China; l No.3 Department of Infectious Diseases, Guanggu District, The Maternal and Child Health Hospital of Hubei Province, Wuhan, Hubei, P. R. China

**Keywords:** COVID-19, asymptomatic, hypertension, early treatment

## Abstract

Novel coronavirus disease 2019 (COVID-19), caused by novel coronavirus SARS-CoV-2, has spread globally since the end of 2019. Asymptomatic carriers are of great concern as they can undermine the interventions to stop the pandemic. However, there is limited information about the characteristics and outcomes of the asymptomatic patients. Therefore, we conducted this retrospective study and retrieved data of 79 asymptomatic COVID-19 patients at admission from three designated hospitals in Wuhan, China. The asymptomatic patients could happen at any age, ranged from 9 to 96 years. These patients also had lower levels of alanine aminotransferase and C-reactive protein. Patchy shadowing was the most common manifestation in computed tomography scan. Some asymptomatic carriers developed mild or moderate symptoms during hospitalization. Age and comorbidities, especially hypertension, may be predictive factors for symptom development in the initially asymptomatic carriers at admission. Early detection and treatment for these presymptomatic patients before symptom onset can shorten the communicable period for the coronavirus and reduce the occurrence of severe cases.

## Introduction

Since December 2019, an outbreak of novel coronavirus disease 2019 (COVID-19), caused by severe acute respiratory syndrome coronavirus 2 (SARS-CoV-2) has spread globally, affecting more than 200 countries and 3,000,000 individuals and causing over 200,000 deaths worldwide until April 30[1]. Although the overall mortality rate of COVID-19 is low, the disease can be transmitted rapidly, and people are generally susceptible to its infection. The virus is primarily spread from person to person by respiratory droplets [2]. The COVID-19 incubation period ranges from 6 to 8 days, and patients can develop symptoms such as fever, cough, myalgia, pneumonia, and even respiratory failure after this period. However, a proportion of the patients are asymptomatic carriers, who are diagnosed based on positive viral nucleic acid test and show no COVID-19 symptoms. The transmission of the novel coronavirus from an asymptomatic carrier with normal chest computed tomography (CT) has been reported [3,4]. A cohort study in China with 44,672 cases reported 1.2% asymptomatic carriers [5], which is believed to be much underestimated. Recent reports suggested that 10–30% of patients infected with SARS-CoV-2 are asymptomatic. Thus, considering that the asymptomatic carriers are infectious, it’s essential to identify them in order to effectively contain the spread of the virus.

Till now, limited data are available for the prevalence and characteristics of the asymptomatic carriers [3], especially the data about the recurrence of positive SARS-CoV2 RNA in these carriers. In addition, some asymptomatic patients can develop symptoms during hospitalization [6], and the characteristics of these presymptomatic patients and their independent risk factors have not been addressed yet. Therefore, in the present study, we investigated 79 asymptomatic patients to analyze their clinical characteristics, disease progression, and recurrence of positive SARS-CoV2 RNA after discharge.

## Methods

### Study design and subjects

**Figure 1.**
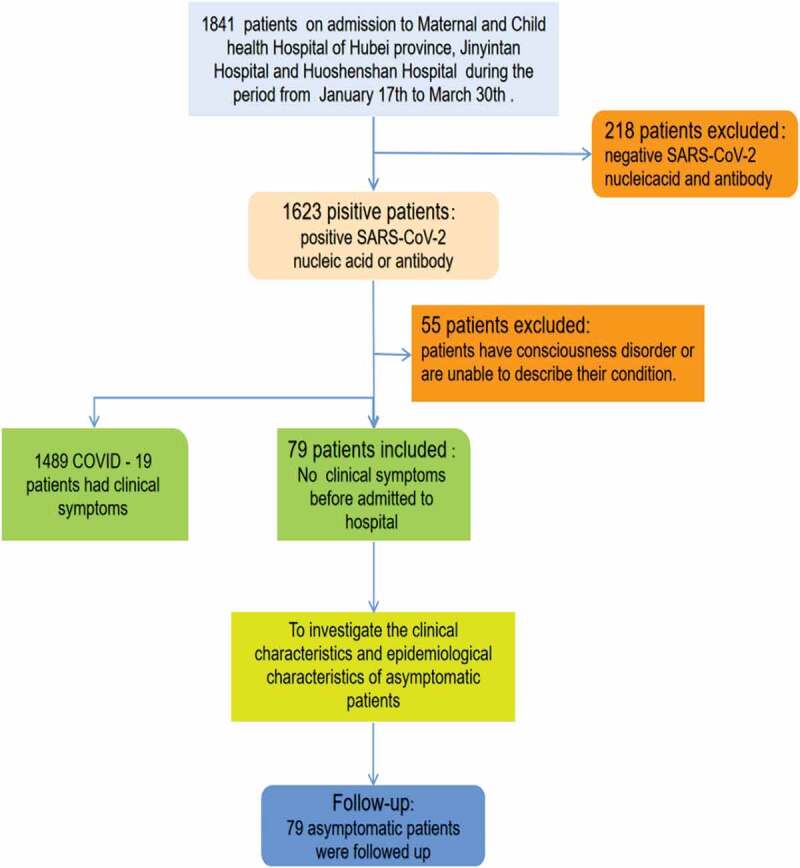
Flow chart of the cohort study.

A total of 1841 patients suspected to be COVID-19 positive and hospitalized between Jan 17 and Mar 30 in three designated hospitals in Wuhan, China (the Maternal and Child Health Hospital of Hubei Province, Wuhan Jinyintan Hospital, and Wuhan Huoshenshan Hospital) were recruited for the study. We analyzed the demographic and clinical characteristics of the COVID-19 patients who were diagnosed according to the guidelines from the National Health Commission of the People’s Republic of China and the interim guidance from World Health Organization interim guidance [7]. After excluding the non-COVID-19 patients, clinically diagnosed patients, unconscious patients, and mental patients, the remaining 1568 patients were divided into two groups based on their symptoms at admission (Figure 1). All discharged COVID-19 patients were isolated and observed in a quarantine facility for two weeks continuously. Seventy-nine asymptomatic cases were recruited, and the final data of follow up was Mar 14 April 2020 or later. All these carriers received anti-viral therapy, traditional Chinese medicine (TCM), and symptomatic treatment during hospitalization. These initially asymptomatic carriers were then further grouped into presymptomatic patients and completely asymptomatic patients according to their disease progression during hospitalization. The study was approved by the Ethics Committee of Naval Hospital of Eastern Theater of PLA and complied with the Declaration of Helsinki. Informed consent for this retrospective study was waived by the ethics committee.

### Data collection

Information regarding epidemiological characteristics, demographic characteristics, underlying comorbidities, clinical symptoms, laboratory findings, chest CT scans, treatments, and prognosis were obtained from the electronic medical records. Laboratory tests and CT scan used data generated at admission. The viral shedding duration was defined as the interval between the first day when a patient had a positive nucleic acid test and the first day when a patient began to have continuous negative tests.

### Statistical analysis

SPSS 20.0 software was applied for statistical analysis. Continuous variables are expressed as the medians and interquartile ranges (IQR). Proportions (categorical variables) were compared by chi-square test with continuity correction or the Fisher’s exact test. Intergroup differences were assessed using Wilcoxon rank-sum test for normally distributed continuous variables and Mann-Whitney U test for skewed continuous variables.

Variables were eligible for entry into a multiple logistic regression model if there were intergroup differences (*P* < 0.05). Logistic regression analysis and the area under the receiver operator characteristic (ROC) curve were used to predict discrimination accuracy. *P* value less than 0.05 indicated a statistically significant difference.

## Results

### Demographic and clinical characteristics of asymptomatic COVID-19 patients

A total of 1568 patients from three designated hospitals were enrolled in the study, including 79 asymptomatic patients (median age: 60.00 years [IQR, 41.00–70.00]; age range: 9–96 years; gender: 32 males and 47 females) and 1489 symptomatic patients (median age: 60.00 years [IQR,49.00–68.00]; age range: 14–100 years). Of the 79 asymptomatic cases, 60 (75.95%) had a history of direct contact with COVID-19 patients in family (37, 46.84%), nursing home (11, 13.92%), workplace (8, 10.12%), and public venues (4, 5.06%).

Asymptomatic infection can occur at any age, but had a higher prevalence in patients aged <45 compared with the symptomatic group (Table 1). Hypertension and diabetes were the most common comorbidities, and incidence rate of diabetes was higher in the asymptomatic group than in the symptomatic group (20.25% vs. 11.69%). The two groups did not differ in the incidence rates of comorbidities including respiratory disease, hypertension, coronary heart disease, chronic renal disease. The asymptomatic group also had more patients without abnormalities in radiographic presentations at admission than the symptomatic group (8.86% vs. 3.83%). No intergroup differences were observed in other radiographic characteristics, and patchy shadowing was the most common abnormality (59.49% vs. 66.15%), and most abnormalities were bilateral (75.95% vs 82.27%) in asymptomatic and symptomatic carriers.

**Table 1.** Demographics and baseline characteristics of the asymptomatic patients with COVID-19.

Characteristics	Value		*P* value
	Asymptomatic patients (*n* = 79)	Symptomatic patients (*n* = 1489)	
**Age**			
Median(IQR) (range)	60.00 (41.00,70.00) (9–96 yr)	60.00(49.00,68.00) (14–100 yr)	0.663
Age distribution (***n***, %)			
<18 yr*	2(2.53)	6(0.40)	0.003
18–45 yr*	23(29.11)	270(18.10)	
46–65 yr*	24(30.38)	712(47.82)	
≥66 yr	30(37.97)	501(33.65)	
Sex (***n***, %)			
Male	32(40.51)	617(41.44)	0.870
Female	47(59.49)	872(58.56)	
Coexisting disorder (***n***, %)			
Respiratory disease	1(1.27)	83(5.57)	0.122
Coronary heart disease	3(3.80)	77(5.17)	0.781
Diabetes	16(20.25)	174(11.69)	0.023
Hypertension	17(21.52)	416(27.94)	0.214
Cerebrovascular disease	4(5.06)	50(3.36)	0.622
Chronic hepatopathy	1(1.27)	22(1.48)	1.000
Chronic renal disease	3(3.80)	15(1.00)	0.058
Cancer	5(6.33)	33(2.22)	0.039
Presence of no less than two comorbidities	14(17.72)	299(20.08)	0.668
Abnormalities on chest CT(***n***, %)			
Unilateral	12(15.19)	207(13.90)	0.077
Bilateral	60(75.95)	1225(82.27)	
No abnormalities*	7(8.86)	57(3.83)	
Ground-glass opacity	19(24.05)	410(27.54)	0.498
Patchy shadowing	47(59.49)	985(66.15)	0.224
Interstitial abnormalities	7(8.86)	65(4.37)	0.113
Thickening of the adjacent pleura	11(13.92)	186(12.49)	0.708
Clinical classification (***n***, %)			
Mild	7(8.86)	57 (3.83)	<0.001
Moderate	70(88.61)	1166(78.31)	
Severe	2(2.53)	217(14.57)	
Critical	0(0)	49(3.29)	
**Clinical outcome**			0.505
Discharged	79(100.00)	1465(98.39)	
Died	0(0)	24(1.61)	

Data are presented as median (IQR) or *n* (%);*P < 0.05 vs. the symptomatic group; IQR: interquartile ranges; COVID-19: novel coronavirus disease 2019; CT: computed tomography.

Patients’ laboratory findings at admission are summarized in Table 2. Compared with the symptomatic patients, the asymptomatic patients had lower levels of monocytes (0.35 × 10^9^/L [IQR0.27,0.45] vs. 0.38 × 10^9^/L [IQR 0.30, 0.47]), alanine aminotransferase (ALT) (15.00 U/L [IQR 10.20, 26.10] vs. 21.00 U/L [IQR 13.70, 34.20]),and C reactive protein (CRP) (0.94 mg/L [IQR 0.40, 2.15] vs. 1.50 mg/L [IQR 0.56, 4.00]). Meanwhile, the asymptomatic carriers had normal levels of other markers related to liver damage, renal dysfunction, inflammation, and coagulation, which were comparable with those of the symptomatic patients.

**Table 2.** Laboratory findings of the asymptomatic patients with COVID-19.

Characteristics	Value		*P* value
	Asymptomatic patients (*n* = 79)	Symptomatic patients (*n* = 1489)	
**Blood routine test**			
Leukocyte (×10⁹/L; normal range 3.50–9.50)	5.60 (4.70,7.10)	5.60 (4.70,6.70)	0.778
Neutrophils (×10⁹/L; normal range 1.80–6.30)	3.39 (2.73,4.63)	3.41 (2.64,4.36)	0.985
Lymphocyte (×10⁹/L; normal range 0.10–3.20)	1.64 (1.32,2.04)	1.60 (1.26,2.00)	0.579
Monocyte (×10⁹/L; normal range 0.10–0.60)	0.35 (0.27,0.45)	0.38 (0.30,0.47)	0.046
Platelet (×10⁹/L; normal range 125.00–350.00)	210.00 (184.00,247.00)	218.00 (179.00,261.00)	0.596
**Blood biochemistry**			
Albumin (g/L; normal range 35.00–52.00)	38.70 (36.43,41.68)	38.80 (36.10,41.10)	0.596
Alanine aminotransferase (U/L; normal range 0.00–55.00)	15.00 (10.20,26.10)	21.00 (13.70,34.20)	<0.001
Aspartate aminotransferase (U/L; normal range 5.00–34.00)	15.20 (12.40,20.10)	16.20 (12.80,22.60)	0.244
Total bilirubin (μmol/L; normal range 0.00–21.00)	9.25 (6.82,13.72)	9.50 (7.40,12.80)	0.764
Blood urea nitrogen (mmol/L; normal range 3.10–8.80)	4.47 (3.72,5.68)	4.56 (3.73,5.59)	0.771
Serum creatinine (μmol/L; normal range 49.00–90.00)	63.90 (54.70,72.10)	63.85 (55.00,75.80)	0.474
Creatine kinase (U/L; normal range 0.00–190.00)	60.00 (44.50,106.75)	56.00 (41.00,79.00)	0.302
Myoglobin (ng/mL; normal range 0.00–146.90)	34.60 (24.88,58.65)	33.50 (25.70,45.10)	0.268
Glucose (mmol/L; normal range 3.89–5.83)	4.64 (4.25,5.89)	4.80 (4.36,5.52)	0.342
**Coagulation function**			
Activated partial thromboplastin time (s; normal range 21.00–37.00)	32.60 (29.95,35.75)	31.70 (29.60,34.10)	0.132
Prothrombin time (s; normal range 9.20–15.00)	11.60 (10.70,12.10)	11.40 (10.90,12.00)	0.938
D-dimer (mg/L; normal range 0.00–0.55)	0.26 (0.15,0.56)	0.34 (0.21,0.77)	0.077
**Infection-related biomarkers**			
Procalcitonin (ng/mL; normal range 0.00–0.050)	0.04 (0.04,0.05)	0.04 (0.04,0.06)	0.149
Erythrocyte sedimentation rate (mm/h; normal range 0.00–15.00)	24.00 (12.00,66.00)	26.00 (12.00,55.00)	0.987
C-reactive protein (mg/L; normal range 0.00–10.00)	0.94 (0.40,2.15)	1.50 (0.56,4.00)	0.019
PLR	98.40 (131.43,162.10)	132.88 (103.25,176.24)	0.414
NLR	2.06 (1.57,2.83)	2.08 (1.58,2.90)	0.636
LMR	4.60 (3.48,6.38)	4.30 (3.24,5.50)	0.071

Data are presented as median (IQR) or n (%). IQR: interquartile ranges; COVID-19: novel coronavirus disease 2019; CT: computed tomography. PLR: platelet-to-lymphocyte ratio; NLR: neutrophil-to-lymphocyte ratio; LMR: lymphocyte-to-monocyte ratio

Though some asymptomatic patients at admission developed symptoms during hospitalization, most of the symptoms were mild or moderate (Table 1). Only 2.53% of asymptomatic carriers developed severe disease, but not critical disease, and all of these patients were discharged from the hospitals. However, 14.57% of the symptomatic patients presented severe or critical disease with a mortality of 3.29%.

### Characteristics of presymptomatic COVID-19 patients

Of the 79 initially asymptomatic patients at admission, 34(43.03%) of them developed symptoms during hospitalization. The median age in these presymptomatic patients was higher than that of the completely asymptomatic patients (68.50 years(IQR 49.00, 80.50) vs. 55.00 years (IQR 37.50,64.00)) (Table 3). The symptoms developed about 8.00 days (median, IQR[5.00,12.00 days], 95%CI 4.36–11.64 days) post positive nucleic acid test. The minimum and maximum incubation period were 2 and 28 days, respectively. Fever (28/34, 82.35%) was the main symptom in these presymptomatic patients (**Supplementary Table 1**). Most of their body temperatures were under 38°C, and only two presented high fever (>38°C). Compared with the symptomatic patients, the presymptomatic patients had less symptoms including lower incidences of cough, fatigue, and chest distress.

**Table 3.** The clinical features of the presymptomatic patients and the completely asymptomatic patients with COVID-19.

	Value		
Characteristics	Presymptomatic patients (*n* = 34)	Completely asymptomatic patients (*n* = 45)	*P* value
**Age**			
Median (IQR) (range)	68.50(49.00,80.50) (16–96 yr)	55.00(37.50,64.00) (9–85 yr)	0.002
Distribution -(n, %)			
<18 yr	1 (2.94)	1 (2.22)	0.008
18–45 yr	7 (20.59)	16 (35.56)	
46–65 yr*	6 (17.65)	18 (40.00)	
≥66 yr*	20 (58.82)	10 (22.22)	
**Sex(female) (n, %)**			0.135
Male	17 (50.00)	15 (33.33)	
Female	17 (50.00)	30 (66.67)	
**Coexisting disorder (n, %)**			
Asthma	0 (0)	1 (2.22)	1.000
Diabetes	10 (29.41)	6 (13.33)	0.078
Hypertension	15 (44.12)	2 (4.44)	<0.001
Coronary heart disease	3 (8.82)	0 (0)	0.076
Cerebrovascular disease	2 (5.88)	2 (4.44)	1.000
Chronic renal disease	3 (8.82)	0 (0)	0.076
Cancer	3 (8.82)	2 (4.44)	0.647
Total with≥2 comorbidity	11 (32.35)	3 (6.67)	0.003
**Abnormalities on chest CT (n, %)**			
Unilateral	7 (20.59)	5 (11.11)	0.185
Bilateral	25 (73.53)	35 (77.78)	
No abnormalities	2 (5.88)	5 (11.11)	
Ground-glass opacity	6 (17.65)	13 (28.89)	0.247
Patchy shadowing	23 (67.65)	24 (53.33)	0.199
Interstitial abnormalities	4 (11.76)	4 (8.89)	0.720
Thickening of the adjacent pleura	6 (17.65)	5 (11.11)	0.615
**Blood routine test**			
Leukocytes (× 10⁹ per L; normal range 3.50–9.50)	5.50 (60,7.20)	5.70 (4.75,7.10)	0.801
Neutrophils (× 10⁹ per L; normal range 1.80–6.30)	3.25 (2.67,4.81)	3.42 (2.75,4.41)	0.972
Lymphocytes (× 10⁹ per L; normal range 1.10–3.20)	1.58 (1.31,1.95)	1.69 (1.31,2.12)	0.461
Monocyte (× 10⁹ per L; normal range 0.10–0.60)	0.39 (0.28,0.48)	0.35 (0.27,0.44)	0.349
Platelets (×10⁹per L; normal range 125.00–350.00)	204.50 (177.25,237.50)	213.00 (187.00,250.00)	0.223
Neutrophil percentage (%; normal range 40.00–75.00)	62.00 (57.50,66.45)	59.00 (52.15,67.85)	0.744
lymphocytes percentage (%; normal range 20.00–50.00)	29.90 (23.45,33.23)	30.70 (23.95,36.50)	0.329
Monocytes percentage (%; normal range 3.00–10.00)	6.15 (4.88,7.55)	6.10 (5.15,7.25)	0.988
**Blood biochemistry**			
Albumin (g/L; normal range 35.00–52.00)	37.75 (35.40,39.28)	40.20 (37.60,41.80)	0.004
Alanine aminotransferase (U/L; normal range0.00–55.00)	14.90 (8.50,21.30)	15.35 (10.30,27.80)	0.309
Aspartate aminotransferase (U/L; normal range5.00–34.00)	16.10 (13.80,23.38)	14.25 (12.10,19.33)	0.090
Total bilirubin (μmol/L; normal range 0.00–21.00)	10.70 (6.13,13.55)	8.80 (7.13,13.95)	0.592
Blood urea nitrogen (mmol/L; normal range 3.10–8.80)	4.58 (3.74,6.27)	4.43 (3.71,5.22)	0.592
Serum creatinine (μmol/L; normal range 49.00–90.00)	67.05 (60.05,80.55)	59.50 (52.80,65.70)	0.008
Glucose (mmol/L; normal range 3.89–5.83)	4.64 (4.28,6.94)	4.55 (4.17,5.28)	0.287
**Infection-related biomarkers**			
C-reactive protein (mg/L; normal range 0.00–10.00)	1.14 (0.50,4.56)	0.89 (0.23,1.92)	0.112
PLR	133.10(99.10,151.22)	129.50 (97.70,167.90)	0.905
NLR	2.10 (1.60,3.00)	1.94 (1.55,2.80)	0.411
LMR	4.17 (3.15,5.11)	5.42 (3.49,7.02)	0.069
**Nucleic acid qPCR test**			
Nucleic acid positive duration	14.00 (7.00,25.00)	12.00 (5.00,24.00)	0.749
Redetected qPCR+	3 (8.82)	5 (11.11)	1.000
**Incubation period**	8.00 (5.00,12.00)		
**Treatment**			
TraditionalChinese medicine	34 (100.00)	45 (100.00)	
antiviral therapy	32 (94.12)	38 (84.44)	0.286

Data are presented as median (IQR) or *n* (%); *P < 0.05 vs. the symptomatic group; IQR: interquartile ranges; COVID-19: novel coronavirus disease 2019; CT: computed tomography; qPCR: quantitative polymerase chain reaction; PLR: platelet-to-lymphocyte ratio; NLR: neutrophil-to-lymphocyte ratio; LMR: lymphocyte-to-monocyte ratio.

Compared with the completely asymptomatic patients, the presymptomatic patients had more comorbidities (52.94% vs. 22.22%), especially hypertension (44.12% vs. 4.44%), and higher incidence of having over two coexisting disorders (32.35% vs. 6.67%). The radiographic characteristics of the presymptomatic patients were similar to those of the asymptomatic patients. The completely asymptomatic patients also had bilateral abnormalities (77.78%), and patchy shadowing (53.33%) was their most common abnormality. They also had lower level of albumin and higher serum creatinine than the presymptomatic patients. The levels of ALT and CRP also differed between these two groups.

### Risk factors for presymptomatic COVID-19 patients

As comorbidity may be an important risk factor of presymptomatic patients, we then focused on the subgroup of the asymptomatic patients with or without comorbidities (**Supplementary Table 2**). Of the asymptomatic patients, those with comorbidities were older (68.50 years vs. 51.00 years) and were more likely to develop symptoms during hospitalization compared with those without comorbidities (64.29% vs. 31.37%). The patients with comorbidities also had higher percentage of bilateral patchy shadowing in chest CT scan, higher levels of blood urea nitrogen and CRP, and lower levels of albumin than the asymptomatic patients without comorbidities. Hypertension and diabetes were the two most common comorbidities. Furthermore, of the 17 asymptomatic patients with hypertension, 11 (64.70%) suffered from two or more comorbidities, and 15 (88.23%) developed symptoms during hospitalization.

To identify the independent risk factors associated with the presymptomatic COVID-19 patients at admission, multivariate logistic regression analysis on laboratory variables and demographic data was conducted (Table 4). At baseline, age and hypertension were independent discriminatory variables and risk factors for presymptomatic patients. A mode (AUC 0.751, 95%CI 0.636–0.866) combining age>69.5 years with hypertension may discriminate presymptomatic patients from completely asymptomatic patients at admission, with a sensitivity of 56.7% and specificity of 87.2%(**Supplementary Table 3, and Supplementary Figure 1**).

**Table 4.** Multivariate analysis of the presymptomatic patients and the completely asymptomatic patients with COVID-19.

Variable	B	SE	Wald	*P* value	OR	OR 95% CI
Age	1.629	0.766	4.528	0.033	5.100	1.137–22.870
Hypertension	2.150	0.854	6.334	0.012	8.583	1.609–45.785

B: partial regression coefficient; SE: standard error: Wald: Wald test statistic: OR: odds ratio; CI: confidence interval; COVID-19: novel coronavirus disease 2019.

### The viral shedding duration and recurrence of asymptomatic carriers

All the asymptomatic patients received antiviral therapy, traditional Chinese medicine, and symptomatic treatment during hospitalization. Although the presymptomatic patients developed symptoms during hospitalization, these patients had similar viral RNA shedding duration compared with the asymptomatic carriers (14 days [IQR 7–25 days] vs. 12 days [IQR 5–24 days])(Table 3). The viral shedding duration in the presymptomatic cases may even be 8 days [IQR 6–15.25 days] since the symptom onset. Surprisingly, the completely asymptomatic patients also had recurrence of positive SARS-CoV-2 RNA after discharge, which was similar to the presymptomatic patients (11.11% vs. 8.82%).

## Discussion

Asymptomatic COVID-19 carriers are estimated to comprise 10–30% of SARS-CoV-2 infected patients. As viral RNA sheds in the upper respiratory tract, it has been proven that both asymptomatic and presymptomatic patients are contagious. Recently, one report revealed that the rates of virus infection through close contact with symptomatic patients and with asymptomatic patients were 6.30% and 4.11%, respectively [8]. Since the asymptomatic patients are more difficult to be identified and isolated than the symptomatic carriers, they might be one of the major drivers of the COVID-19 pandemic [9].

In this study, we reported 79 laboratory-confirmed asymptomaticCOVID-19 cases. We found that younger carriers were more prone to be symptom-free. However, diabetes and cancer were more common in the asymptomatic patients, which was different from the previous reports that comorbidities were mainly the risk factors for severe COVID-19. After analyzing these asymptomatic patients, we found that 11 cases in the asymptomatic group were from the same nursing home coronavirus cluster. Elevated occurrence of diabetes and cancer in these elder patients caused increased incidence of diabetes and cancer in the asymptomatic group. In addition, most of these patients (7/11, 63.64%) were actually presymptomatic patients who developed symptoms during hospitalization. Other comorbidities were not correlated with the development of symptomatic or asymptomatic cases, indicating common comorbidities may have no significant relevance in distinguishing those asymptomatic carriers at admission.

The radiographic abnormalities of the asymptomatic patients were almost similar to those of the symptomatic patients in our study, except for the fact that the asymptomatic group had slightly more patients without abnormalities (8.86% vs. 3.83%), indicating CT scan can not identify the asymptomatic carriers. However, a recent paper reported nearly half (43.2%) asymptomatic patients without radiographic abnormalities [10]. In addition, they also found most of the abnormalities were unilateral, which is inconsistent with our findings. Thus, the exact radiographic characteristics of asymptomatic patients needs further investigation.

Though some of the asymptomatic cases developed symptoms during hospitalization, most of these cases were mild or moderate. The lower levels of ALT and CRP in the asymptomatic patients compared with symptomatic patients indicated these patients had less liver damage and inflammation. Some studies demonstrated a substantial decrease in the total number of lymphocytes in COVID-19 patients. However, no marked reduction of lymphocytes were observed in both asymptomatic and symptomatic patients in our study. Reduced lymphocytes may indicate a more severe phenotype of the disease. The severely diseased COVID-19 patients comprised a less percent in the symptomatic group, and some of their conditions were even moderate or mild at admission. Thus, the decreased lymphocytes may be observed with further detailed classification of the symptomatic patients. Although most of the presymptomatic patients developed mild or moderate disease, two patients developed severe disease and required oxygen therapy or other interventions. Therefore, identifying these severe diseased presymptomatic patients can have great clinical value and more studies need to be carried out in this area.

The presymptomatic patients developed symptoms 8 days post positive nucleic acid test, indicating an incubation period of about 8 days, or even longer considering the interval between SARS-CoV-2 exposure and nucleic acid test. However, previous reports declared a 5–6 day median incubation period from virus exposure [11,12]. Studies also found that the elders had a longer incubation period and a larger variance than the youngers [12]. Thus, the prolonged incubation period found in our study may be due to the higher percentage of the elderly patients and the medical treatment in the presymptomatic group.

To discriminate the presymptomatic patients from the asymptomatic carriers, we also compared them with the completely asymptomatic carriers. Those who were older and had hypertension or combined comorbidities were more likely develop symptoms during hospitalization. Hypertension has been identified previously as a risk factor for severe COVID-19 disease and can cause higher mortality [13,14]. Angiotensin-converting enzyme inhibitor (ACEIs) and angiotensin II receptor blockers (ARBs) have been used in the first-line treatment for hypertension. ACEIs and ARBs can increase the expression level of ACE2, the main cellular entry receptor for SARS-CoV-2 virus. However, a recent retrospective, multicenter study revealed that the hospitalized patients who received ACEI/ARB had lower mortality rate compared with ACEI/ARB nonusers [15]. Thus, presence of hypertension and no usage of ACEI/ARB may be correlated with the disease progression in the asymptomatic carriers.

Albumin and creatinine levels can reflect the function of the liver and kidney. All the asymptomatic patients possessed normal range albumin and creatinine, and the presymptomatic patients had lower level of albumin and higher level of serum creatinine compared with completely asymptomatic patients, suggesting slight liver and kidney injury. This result aligned with the previous reports that the SARS-CoV-2 virus can invade liver and kidney and have significant cytotoxicity toward these cells [16–18]. These correlations also suggested the association between symptoms and organ injury.

The viral shedding duration of the completely asymptomatic patients were 12 days (median), which was comparable to that in the presymptomatic patients. The viral shedding duration of the asymptomatic patients was shorter than that of the symptomatic patients, which was 17 days or even longer [19–22]. In this study, the viral shedding duration was calculated from the first day when a patient had a positive nucleic acid test, which may be underestimated compared with those in symptomatic patients starts from the symptom onset. However, the viral shedding duration of the presymptomatic patients in our study started before symptom onset and was overestimated compared with previous reports. By receiving antiviral therapy and traditional Chinese medicine treatment before the symptom onset, the patients could have a shorter communicable period compared with those symptomatic patients in the previous studies [22]. This finding strongly indicated that early detection and early treatment of the presymptomatic patients can not only stop the rapid transmission but also reduce the viral communicable period. Another study also demonstrated that delayed admission to hospital after illness onset is associated with prolonged viral shedding duration [22]. In addition, early treatment may also reduce the severe disease ratio, as relatively fewer presymptomatic patients in our study developed severe or critical disease compared with the symptomatic patients who started treatment after symptom onset. However, a recent paper reported a median duration of viral shedding in the asymptomatic group as 19 days [10]. These may be caused by the resource of the patients, as their patients were from active screening close contacts under quarantine, but ours were from those who came to hospital by themselves.

The asymptomatic carriers may also be redetected positive for SARS-CoV-2 RNA after discharge, even only had mild disease and no abnormalities in the laboratory parameters. This finding was consistent with the report that mild diseased patients are more prone to be redetected positive after discharge [23,24]. In our study, the recurrence rate of positive SARS-CoV-2 nucleic acid in the asymptomatic carriers after discharge was lower than that in the symptomatic patients (10.12% vs. 14.5%) [24]. Although the exact reasons for this recurrence remain unclear, false negativity, residual virus, and age distribution may be potential factors [25,26]. Recently, postmortem pathological evidence revealed the presence of residual virus in the pulmonary tissues of patient who had a sudden death when ready for discharge [27]. These explanations for symptomatic patients may also apply to the asymptomatic carriers. Therefore, in addition to the asymptomatic coronavirus transmission, the recurrence in the asymptomatic carriers should also be taken into concern to control the spread of the virus. The characteristics of these asymptomatic patients with recurrence of positive SARS-CoV-2 RNA require further investigation with a mass of samples.

There were still limitations to this study. First, only 79 asymptomatic inpatient cases were included, and most of the asymptomatic patients were under medical observation in mobile cabin hospitals or even in the open. A more extensive study on these cases need to be conducted to describe their characteristics and find independent risk factors for the disease progression. Furthermore, since only two asymptomatic cases progressed to severe disease during hospitalization, no clues were found on how to distinguish these asymptomatic patients who may develop severe disease. In addition, since we did not have adequate information about the levels of inflammatory cytokines in these cases, we could not analyze whether the inflammatory state of the asymptomatic carriers was correlated with the progression and severity of the disease in these asymptomatic patients. Therefore, large-scale retrospective or prospective studies with more detailed information are needed to expand and validate our study.

In sum, our study has been the largest multicenter retrospective study on asymptomatic COVID-19 carriers to describe their clinical characteristics and their disease progression. Patients under 45 years old are more likely to develop asymptomatic infection. Age and presence of hypertension may be predictive factors for the symptomatic progression of asymptomatic carriers at admission. Recurrence of positive SARS-CoV-2 nucleic acid may also happen in asymptomatic carriers. Early detection and treatment of these asymptomatic patients can shorten the communicable period and reduce the chances to develop severe disease. As a result, a more scientific control strategy with more focus on these asymptomatic patients need to be developed to control the COVID-19 pandemic.

## Supplementary Material

Supplemental Material

Supplemental Material
