# Supplementary material for: Characteristics of asymptomatic COVID-19 infection and progression: A multicenter, retrospective study
Source: Virulence. 2020 Aug 12;11(1):1006–14. doi: 10.1080/21505594.2020.1802194 (PMC7550018; doi:10.1080/21505594.2020.1802194)

**Supplementary information for**

**Characteristics of asymptomatic COVID-19 infection and progression: a multicenter, retrospective study**

Chao Yu*^1,9^, Miao Zhou*^2^, Yang Liu*^3^, Tinglin Guo^1,9^, Chongyang Ou^1,9^, Liye Yang^3,10^, Yan Li^4,11^, Dongliang Li^5,8^, Xinyu Hu^6^, Li Shuai^1^, Bin Wang^7^, Zui Zou^3,12^

**Supplementary Table 1. The symptoms of** **the pre-symptomatic and the symptomatic patients with COVID-19**

| **Characteristics** | **Value** | | **P value** |
| --- | --- | --- | --- |
|  | **Pre-symptomatic patients(n=34)** | **Symptomatic patients(n=1489)** |  |
| **Signs and symptoms (n, %)** |  |  |  |
| Fever | 28(82.35) | 949(63.73) | 0.025 |
| Cough | 10(29.41) | 860(57.76) | 0.001 |
| expectoration | 1(2.94) | 147(9.87) | 0.291 |
| Sore throat | 1(2.94) | 59(3.96) | 1.000 |
| Shortness of breath | 4(11.76) | 248(16.66) | 0.640 |
| Fatigue | 4(11.76) | 530(35.59) | 0.003 |
| Chest distress | 0(0) | 219(14.71) | 0.030 |
| [diarrhea](../../AppData/Local/youdao/dict/Application/8.9.0.0/resultui/html/index.html#/javascript:;) | 1(2.94) | 40(2.69) | 0.575 |
| Anorexia | 3(8.82) | 93(6.25) | 0.470 |
| **Highest temperature during hospitalization (n, %)** |  |  |  |
| <37.3°C | 6(17.65) | 1112(74.68) | <0.001 |
| 37.3–38.0°C | 26(76.47) | 335(22.50) |  |
| 38.1–39.0°C | 1(2.94) | 33(2.22) |  |
| >39.0°C | 1(2.94) | 9(0.60) |  |

Data are presented as median (IQR) or n (%); IQR: interquartile ranges; COVID-19: novel coronavirus disease 2019.

**Supplementary Table2.The impact of coexisting disorders on the clinical characteristics of the asymptomatic patients with COVID-19**

| **Characteristics** | **Value** | | **P value** |
| --- | --- | --- | --- |
|  | **Patients with coexisting disorder（n=28）** | **Patients without coexisting disorders（51）** |  |
| **Age** |  |  |  |
| Median(IQR) (range) | 68.50 (56.50,75.75)  (40-96yr) | 51.00(35.00,66.00)(9-91yr) | 0.001 |
| Distribution (n, %) |  |  |  |
| <18yr | 0(0) | 2（3.92) | 0.002 |
| 18-45yr* | 2(7.14) | 21（41.18) |  |
| 46-65yr | 10(35.71) | 14（27.45) |  |
| ≥66yr* | 16(57.15) | 14（27.45) |  |
| **Sex(female) (n, %)** |  |  | 0.080 |
| Male | 15(53.57) | 17(33.33) |  |
| Female | 13(46.43) | 34(66.67) |  |
| **Group** |  |  |  |
| Pre-symptomatic patients | 18(64.29%） | 16（31.37%) | 0.005 |
| Asymptomatic carrier | 10(35.71%） | 35（68.63%) |  |
| **Abnormalities on chest CT (n, %)** |  |  |  |
| Unilateral | 2(7.14) | 10(19.61) | 0.022 |
| Bilateral^*^ | 26(92.86) | 34(66.67) |  |
| No abnormal^*^ | 0(0) | 7(13.72) |  |
| Ground-glass opacity | 3(10.71) | 16(31.37) | 0.054 |
| patchy shadowing | 23(82.14) | 24(47.06) | 0.004 |
| Interstitial abnormalities | 3(10.71) | 5(9.80) | 1.000 |
| Thickening of the adjacent pleura | 6(11.76) | 5(9.80) | 0.184 |
| **Blood routine test** |  |  |  |
| Leukocytes (× 10⁹ per L; normal range 3.50-9.50) | 5.50(4.73,7.10) | 5.80(4.60,7.10) | 0.902 |
| Neutrophils (× 10⁹ per L; normal range 1.80-6.30) | 3.21(2.66,4.26) | 3.49(2.73,4.81) | 0.479 |
| Lymphocytes(× 10⁹ per L; normal range 1.10-3.20) | 1.69(1.29,2.16) | 1.58(1.32,2.00) | 0.663 |
| monocyte(× 10⁹ per L; normal range 0.10-0.60) | 0.36(0.27,0.43) | 0.35(0.26,0.45) | 0.806 |
| Platelets (× 10⁹ per L; normal range 125.00-350.00) | 217.50(181.75,280.00) | 210.00(186.00,245.00) | 0.818 |
| neutrophil percentage  (%; normal range 40.00-75.00) | 62.45(57.00,69.45) | 59.80(52.40,65.60) | 0.131 |
| lymphocytes percentage  (%; normal range 20.00-50.00) | 29.90(21.65,32.48) | 30.70(24.10,36.30) | 0.186 |
| Monocytes percentage  (%; normal range 3.00-10.00) | 5.55(4.80,7.43) | 6.50(5.40,7.30) | 0.129 |
| **Blood biochemistry** |  |  |  |
| Albumin (g/L; normal range35.00-52.00) | 37.50(34.70,40.10) | 39.40(37.60,42.00) | 0.001 |
| Alanine aminotransferase  (U/L; normal range0.00-55.00) | 14.60(10.15,21.15) | 15.10(10.25,27.70) | 0.379 |
| Aspartate aminotransferase  (U/L; normal range5.00-34.00) | 14.20(12.30,20.20) | 15.60(12.75,20.00) | 0.664 |
| Total bilirubin (μmol/L; normal range 0.00-21.00) | 8.70(6.80,13.30) | 11.00(6.85,14.20) | 0.359 |
| Blood urea nitrogen  (mmol/L; normal range 3.10-8.80) | 5.18(3.83,6.45) | 4.18(3.71,5.29) | 0.046 |
| Serum creatinine  (μmol/L; normal range 49.00-90.00) | 65.85(53.98,80.55) | 53.40(56.80,67.10) | 0.242 |
| Glucose (mmol/L; normal range 3.89-5.83) | 5.60(4.48,10.83) | 4.36(4.06,5.05) | 0.001 |
| **Infection-related biomarkers** |  |  |  |
| C-reactive protein (mg/L; normal range 0.00-10.00) | 1.63(0.60,10.85) | 0.72(0.33,1.38) | 0.009 |
| PLR | 131.18(94.47,164.23) | 131.43(99.47,162.10) | 0.967 |
| NLR | 1.93(1.50,2.71) | 2.07(1.58,2.85) | 0.539 |
| LMR | 4.32(3.03,6.06) | 4.74(3.50,6.4) | 0.652 |
| **Nucleic acid qPCR test** |  |  |  |
| Nucleic acid positive duration | 15.00(7.25,25.00) | 12.50(5.00,25.00) | 0.446 |
| Re-detected qPCR+ | 5(17.86) | 3(5.88) | 0.124 |

Data are presented as median (IQR) or n (%); ^*^P<0.05 vs. the symptomatic group； IQR: interquartile ranges; COVID-19: novel coronavirus disease 2019; CT: computed tomography; qPCR: quantitative polymerase chain reaction; PLR: platelet-to-lymphocyte ratio; NLR: neutrophil-to-lymphocyte ratio; LMR: lymphocyte-to-monocyte ratio.

**Supplementary Table 3. Analysis of the predictive factors for the pre-symptomatic patients and the completely asymptomatic patients** **with COVID-19**

| **Variable** | **AUROC** | **Cut-off Value** | **Sensitivity** | **Specificity** | **95%CI** |
| --- | --- | --- | --- | --- | --- |
| Age | 0.691 | 69.500 | 0.471 | 0.911 | 0.598-0.814 |
| Hypertension | 0.698 | 0.500 | 0.441 | 0.956 | 0.576-0.821 |
| Age + hypertension | 0.751 |  | 0.567 | 0.872 | 0.636-0.866 |

AUROC: area under the subject operating characteristic curve; CI: confidence interval; COVID-19: novel coronavirus disease 2019.

**Supplementary Figure1: ROC analysis of the risk factors for the pre-symptomatic patients using logistic regression analysis.**

**ROC: receiver operating characteristic.**


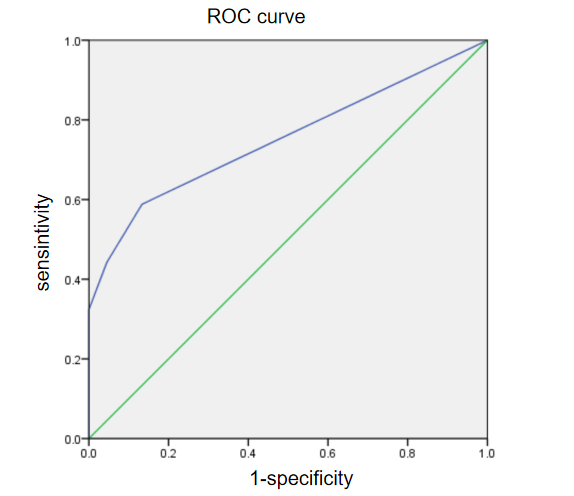

Supplement: Supplemental Material [file KVIR_A_1802194_SM4681.doc]
