# Supplementary figures and images for: Characteristics of asymptomatic COVID-19 infection and progression: A multicenter, retrospective study
Source: Virulence. 2020 Aug 12;11(1):1006–14. doi: 10.1080/21505594.2020.1802194 (PMC7550018; doi:10.1080/21505594.2020.1802194)

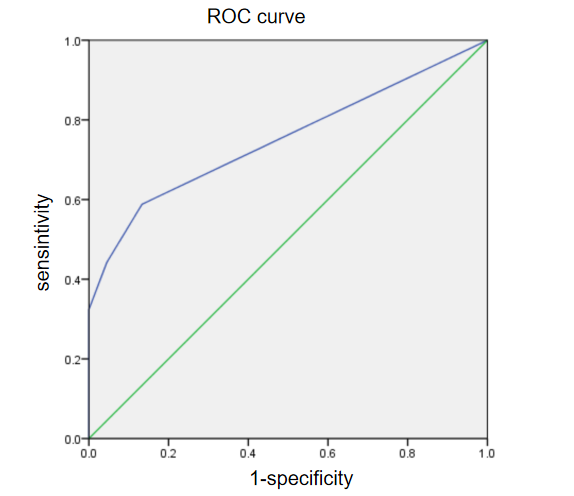

Supplement: Supplemental Material [file KVIR_A_1802194_SM4680.png]
